# Barriers and facilitators to patient-centred care in pharmacy consultations: A qualitative study with Malaysian hospital pharmacists and patients

**DOI:** 10.1371/journal.pone.0258249

**Published:** 2021-10-07

**Authors:** Yew Keong Ng, Noraida Mohamed Shah, Ly Sia Loong, Lay Ting Pee, Wei Wen Chong

**Affiliations:** 1 Faculty of Pharmacy, Centre of Quality Management of Medicines, Universiti Kebangsaan Malaysia, Jalan Raja Muda Abdul Aziz, Kuala Lumpur, Malaysia; 2 Department of Pharmacy, Universiti Kebangsaan Malaysia Medical Centre, Jalan Yaacob Latif, Bandar Tun Razak, Kuala Lumpur, Malaysia; 3 Department of Pharmacy, Hospital Kuala Lumpur, Ministry of Health, Jalan Pahang, Kuala Lumpur, Malaysia; Hong Kong Polytechnic University, HONG KONG

## Abstract

**Background:**

Patient-centred care (PCC) has been suggested to provide benefits such as improved patient-healthcare provider communication and better disease self-management to patients. The practice of PCC should involve all healthcare professionals, including pharmacists who are well-positioned in providing pharmaceutical care to patients. However, a better understanding of the factors that can affect the practice of PCC in pharmacists’ consultations is needed.

**Objective:**

To explore the perceptions of Malaysian hospital pharmacists and patients on the barriers and facilitators of a PCC approach in pharmacist consultations.

**Design:**

This study employed a qualitative, explorative semi-structured interview design.

**Setting and participants:**

Interviews were conducted with 17 patients and 18 pharmacists from three tertiary hospitals in Malaysia. The interviews were audiotaped and transcribed verbatim. Emerging themes were developed through a constant comparative approach and thematic analysis.

**Results:**

Three themes were identified in this study: (i) patient-related factors (knowledge, role expectations, and sociocultural characteristics), (ii) pharmacist-related factors (personalities and communication), and (iii) healthcare institutional and system-related factors (resources, continuity of care, and interprofessional collaboration). Pharmacists and patients mentioned that factors such as patients’ knowledge and attitudes and pharmacists’ personality traits and communication styles can affect patients’ engagement in the consultation. Long waiting time and insufficient manpower were perceived as barriers to the practice of PCC. Continuity of care and interprofessional collaboration were viewed as crucial in providing supportive and tailored care to patients.

**Conclusion:**

The study findings outlined the potential factors of PCC that may influence its implementation in pharmacist consultations. Strategic approaches can be undertaken by policymakers, healthcare institutions, and pharmacists themselves to address the identified barriers to more fully support the implementation of PCC in the pharmacy setting.

## Introduction

Patient-centred care (PCC) is a dynamic and individualised approach to healthcare [1]. PCC represents a shift from traditional biomedical and disease-focused practice to a more holistic approach whereby patients are at the centre of care [2]. This approach focuses on individualised and goal-oriented healthcare planning that considers the patient’s views and preferences. Barry and Edgman-Levitan (2012) highlighted that the active engagement of patients is particularly important in healthcare decision-making, including medication-related decisions [3].

The practice of PCC should involve all healthcare professionals, including pharmacists. Pharmacists are well-versed in medication counselling, which along with their clinical expertise makes them strategically positioned to provide pharmaceutical care to patients. Moreover, studies on pharmacists integrating PCC into their practice have suggested improved outcomes, such as increased medication adherence, better health communication, and a more effective patient-provider relationship [4,5]. Patients are also more involved in consultations, particularly regarding their treatment plans, and are more motivated to self-manage their chronic illnesses [6,7].

In Malaysia, hospital pharmacists are directly involved in medication management and chronic disease monitoring through medication therapy adherence clinic (MTAC) services. MTAC services are performed in a structured manner to identify and address medication-related issues, such as medication non-adherence [8]. Pharmacists also provide medication counselling and educational booklets (e.g. diabetes and warfarin information booklets) to facilitate self-education and enable disease self-management [9]. In addition, pharmacists work together with other healthcare team members, such as physicians, to discuss with patients about their progress and propose additional therapeutic plans. Some examples of these plans include lifestyle modifications and dose adjustments for warfarin based on their International Normalised Ratio (INR) values or insulin therapy based on their self-monitored blood glucose levels or glycated haemoglobin (haemoglobin A1c level) [10,11]. As MTAC pharmacists are directly involved in providing pharmaceutical care to patients, this provides an opportunity for them to practice PCC in their patient encounters. A previous study in the MTAC setting indicated that both pharmacists and patients generally supported a PCC approach [12]. Moreover, MTAC patients who felt actively involved in their consultation had higher self-efficacy, especially in medication decision-making, thereby further establishing the need for a strategy to integrate PCC into pharmacist consultations [13].

The integration of PCC into healthcare consultations remains a challenge in various practice settings. For instance, in a multilingual Malaysian society, language barriers between providers and patients may lead to difficulties in patient engagement and a PCC approach [14]. Patients’ preference for a passive role in healthcare consultations and poor health literacy have been shown to impact the patient-centred approach as well, particularly in shared decision-making [15,16]. In addition, factors, such as time constraints, could hinder the effective application of PCC in encounters [17]. Thus, the application of PCC can be influenced by various barriers and facilitators, highlighting the need to identify and address these factors in specific practice settings, including MTAC. However, there are limited studies on the barriers and facilitators of a PCC approach in the Malaysian healthcare system, especially in pharmacists’ consultations. Therefore, this study aimed to explore the perceptions of Malaysian hospital pharmacists and patients on factors that can hinder or facilitate a PCC approach in MTAC consultations.

## Materials and methods

### Study design and participants

This qualitative study comprised semi-structured interviews with MTAC pharmacists and patients to explore their personal views and experiences on the barriers and facilitators that could affect the implementation of a PCC approach in medication consultations. This study design was selected to gain insights and an in-depth understanding of real-life experiences in the hospital pharmacy setting that may not be fully captured in a quantitative study. In addition, semi-structured interviews allow interviewees to express their opinions on their own terms [18]. Some data from this study regarding the concept and important aspects of patient-centredness in pharmacist consultations were reported previously [12]. Despite being from the same dataset, the current paper reports on a different part of the interviews, which were pharmacists’ and patients’ personal views and experiences on the potential or actual barriers and facilitators of a PCC approach in MTAC consultations. Accordingly, it should be noted that data were retrieved from the interviewees who participated in the previous study [12]. Both pharmacists and patients were recruited to obtain views from multiple perspectives.

Pharmacists were recruited using purposive sampling based on their expertise and experience in various chronic disease management practices in MTAC services. Pharmacists were eligible to participate if they were 1) fully registered as a pharmacist and 2) currently involved in the provision of MTAC services. Patients were recruited by convenience sampling if they fulfilled the following criteria: 1) aged 18 years or older, 2) able to speak and understand English and/or Malay language, and 3) have been on follow-up with MTAC services for at least 2 months.

This study was approved by the Human Research Ethics Committee of Universiti Kebangsaan Malaysia (Ref No: PPI/111/8/JEP-2017-354) and the Medical Research and Ethics Committee, Ministry of Health Malaysia (Ref No: NMRR-17-1471-36916 (IIR)). The study was conducted in one teaching hospital and two tertiary hospitals in Malaysia. Prior to the interview session, the purpose of the interview was explained to the participants and written informed consent was then obtained from all participants.

### Data collection

Interview guides for pharmacists and patients were developed based on the PCC integrative framework by Scholl et al. [19] (see Table 1 for examples of questions). The framework comprised three inter-related domains, namely, principles, activities, and enablers, that contribute to patient-centredness in healthcare consultations. The principles emphasised the core aspects of PCC that included providers equipped with related characteristics (e.g. empathy and integrity), recognising patients as unique individuals, and cultivating mutual understanding. These principles should be applied during patient-centred activities, such as providing tailored information, encouraging patient involvement in decision-making, and supporting patients’ emotional needs. To support these activities, enablers, such as good verbal and non-verbal communication, contribute to operationalizing PCC during consultation [19]. The interview guide and findings related to pharmacists’ and patients’ views regarding the concept of PCC, its essential aspects, and relevance in medication consultations have been reported previously [12]. Responses specific to factors that can facilitate or hinder a PCC approach in pharmacist consultations are presented in this article. Patients were prompted if further clarity was required to ensure that their answers were reflective of their personal experiences in the MTAC consultation.

**Table 1:** Examples of interview questions.

PCC domains^a^	Examples of questions
Principles	
Essential characteristics of the clinician Clinician-patient relationship Patient as unique person Biopsychosocial perspective	Pharmacist questions: • What do you think are the ideal characteristics of a patient? • What elements would be necessary for care to be patient-centred, from your perspective? • What do you think are some challenges in building a therapeutic relationship with patients in MTAC?
	Patient questions: • In your opinion, what do you think are the ideal characteristics of a pharmacist? • What is the most important thing for you in a pharmacist consultation that places you at the centre of care?
Enablers	
Communication Teamwork and team building Coordination and continuity of care	Pharmacist questions: • How are communications between you and the patients reflective of patient-centred care? • How do you check the patient’s understanding of the information given during the MTAC consultation? • What are some things that a multidisciplinary healthcare team can do to facilitate the practice of patient-centred care?
	Patient questions: • Do you feel that you are part of the consultation? (E.g. being able to voice out concerns, speak uninterrupted and be listened to). • What is the most challenging part in the consultation in your opinion? • What do you think hinders patient-centred care or a consultation that puts patients at the centre of care? • How do you think pharmacists can help you to become more active in the consultation?
Activities	
Patient involvement Involvement of family and friends Patient empowerment Emotional support	Pharmacist questions: • What are the types of decisions that are frequently made during the MTAC counselling sessions? • Do you think patients are capable of making their own medication decisions? How can pharmacists support patients in making medication decisions? • What do you think are some challenges in increasing patient involvement in decision-making in pharmacist consultations?
	Patient questions: • How do you think patients can help themselves to be more active in consultations? What forms of support would be useful? • In any event that you may have a question or concerns about your medication, how do you voice them to the pharmacist? • Do you think patients and their caregivers have a role in making treatment-related decisions? How do you think they should participate in treatment decision-making?

^a^PCC domains are based on the framework by Scholl et al.[19].

Data collection for this study was performed from September 2017 to July 2018. Interviews with the pharmacists were conducted in their office rooms or cubicles while ensuring sufficient privacy. On the other hand, patients were taken to a vacant consultation room or a secluded corner in the waiting area to ensure adequate privacy and minimal distraction. All interviews were audiotaped and transcribed verbatim. Field notes were also taken for each interview to support data interpretation [20]. Credibility in this study was established through sufficient time spent by the interviewer (Y.K.N.) to ensure familiarisation with the environment and practice setting of each site. A pilot study was also conducted prior to the actual data collection to test and refine the interview questions and the overall interview process. Interviews were conducted in both English and Malay languages. Interviews conducted in the Malay language were first transcribed, translated into English, and reviewed by bilingual research members to ensure clarity and accuracy. Interviews were conducted until data saturation was attained. Data saturation was achieved when no new themes or codes (also known as ‘informational redundancy’) emerged from the last three interviewees from pharmacists and patients, respectively [21].

### Data analysis

Interview transcripts for both pharmacists and patients were analysed using the thematic analysis method described by Braun and Clarke [22]. The following steps were performed in the analysis: First, transcripts were repeatedly read to ensure familiarisation with the data. Thereafter, initial codes were produced by Y.K.N. and W.W.C., and emerging themes and subthemes were subsequently generated based on significant patterns in the codes. New codes were then continually defined, and themes and subthemes were continuously reviewed and refined using the constant comparison approach before being included in the final write-up [22,23]. In addition, field notes were used to aid early interpretation. Reflexivity was employed by the researchers during the interviews and analyses through memos obtained during and after the interviews. This allows for the critical reflections of feelings and performance among researchers that support their reflexivity and the interpretation of the data [20]. Atlas.ti (version 7) was used to manage the data. The resulting themes and subthemes were discussed with all members of the research team to ensure that a consensus was reached.

## Results

**Fig 1:**
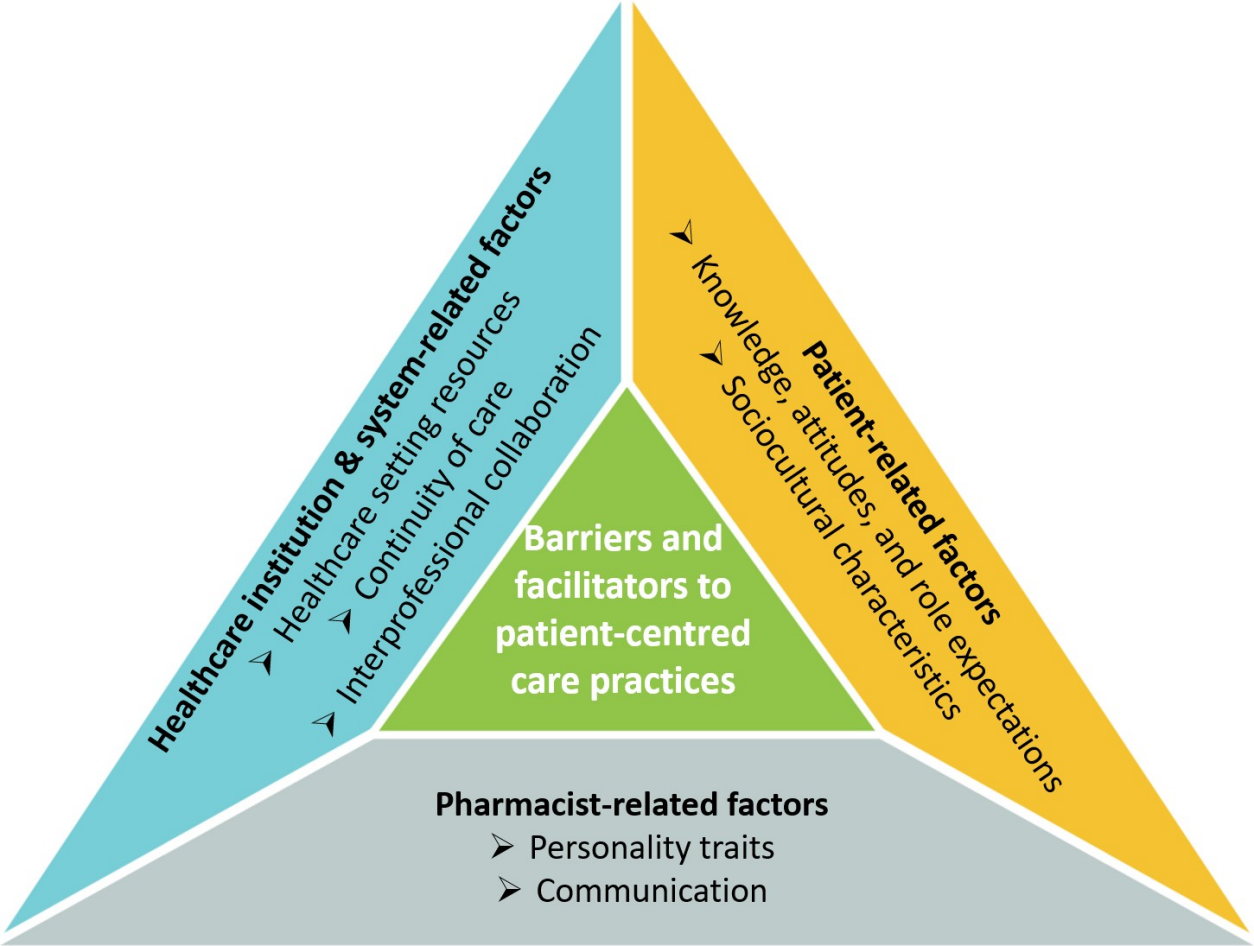
Barriers and facilitators to patient-centred care practice.

Eighteen pharmacists and 17 patients were interviewed. The duration of the interviews ranged from 15 to 55 min. The average duration of interviews for pharmacists and patients was approximately 29 and 25 min, respectively. Tables 2 and 3 show the demographics of pharmacists and patients, respectively. Factors that could facilitate or hinder a PCC approach in pharmacist consultations were divided into three main themes: patient-related factors, pharmacist-related factors, and healthcare institutional and system-related factors. Themes and their respective subthemes are summarised below (Fig 1).

**Table 2:** Demographic characteristics of pharmacists.

Descriptive characteristics	Number (%) (N = 18)
Gender	
Male	1 (5.5)
Female	17 (94.4)
Age (years old)	
25–34	14 (77.8)
35–44	4 (22.2)
Ethnicity	
Malay	2 (11.1)
Chinese	13 (72.2)
Indian	3 (16.7)
Highest academic qualification	
Bachelor’s degree	12 (66.7)
Master’s degree	5 (27.8)
PhD	1 (5.6)
Type of MTAC involved^a^	
Warfarin	8 (44.4)
Diabetes Mellitus (DM)	5 (27.8)
Human Immunodeficiency Virus (HIV)	2 (11.1)
Respiratory	1 (5.6)
Thalassemia	1 (5.6)
Psychiatry	1 (5.6)
Years of experience as practicing pharmacist	
<5 years	3 (16.7)
5–10 years	9 (50.0)
>10 years	6 (33.3)
Hours spent in MTAC consultation per week	
<5 hours	13 (72.2)
5–10 hours	5 (27.8)

^a^MTAC: Medication Therapy Adherence Clinic.

**Table 3:** Demographic characteristics of patients.

Descriptive characteristics	Number (%) (N = 17)
Gender	
Male	8 (47.1)
Female	9 (52.9)
Age (years old)	
18–24	2 (11.8)
25–34	1 (5.9)
35–44	1 (5.9)
45–54	3 (17.6)
55–64	5 (29.4)
Above 65	5 (29.4)
Ethnicity	
Malay	11 (64.7)
Chinese	3 (17.7)
Indian	3 (17.7)
Education level	
Primary school	2 (11.8)
Secondary school	11 (64.7)
Diploma/Pre-university	2 (11.8)
University (Degree, Postgraduate)	2 (11.8)
Monthly personal income^a^	
Not working/retired	13 (76.5)
<RM2,000	3 (17.6)
RM5,001- RM8,000	1 (5.9)
Years of follow-up	
< 6 months	2 (11.8)
6–12 months	1 (5.9)
>1 year	14 (82.3)
Reason for MTAC involvement^b^	
Warfarin	14 (82.3)
DM	2 (11.8)
Hematology	1 (5.9)

^a^Monthly personal income in Malaysian Ringgit currency (RM), median income = RM5,873 in 2019 [24].

^b^MTAC: Medication Therapy Adherence Clinic.

### Patient-related factors

#### Knowledge, attitudes, and role expectations

This factor is related to how the patients’ level of knowledge and intrinsic role expectations could influence their level of involvement in MTAC consultation. Both pharmacists and patients perceived that the lack of knowledge among patients about their health conditions and treatments contributed to patients playing a passive role during consultations. This lack of knowledge was believed to be influenced by the patient’s background, including the level of education.

Especially in the group of patients who may not have that level of understanding to give you feedback, to express what’s their preference or what’s their expectation. It may depend on patients’ background, educational background, and the type of job they’re doing as well. Some may just take in whatever you said; they may not know what to ask to begin with. (Pharmacist 3)

Pharmacists also added that patients who were more knowledgeable about their disease and medications tended to be more active in the encounter.

[For] some of the educated patients, they know something about their disease or what is the treatment for them, [so] usually they will ask more (questions). So, there is more two-way communication. But for some elderly patients, they [had] not really received a lot of education. So usually they will just listen to what the healthcare provider says. (Pharmacist 17)

In this respect, patients also commented that a lack of medical knowledge or expertise may contribute to them making unrealistic or medically inappropriate demands.

[I prefer a] more passive type of role because I am not well-educated in this medical field, I may tend to give the wrong thing (input), or demand [for the] wrong things…if you want to be actively involved in this, that person should have at least some medical knowledge… So, I feel it is quite challenging. (Patient 3)

Several pharmacists suggested that the passive attitude of some patients could be due to patients’ expectation to play a passive role in consultations while expecting healthcare providers to provide the instructions to be followed.

I would say [that] the Malaysian population, those who come to a government hospital, may not be aware that they can actually voice their opinions. I think it’s the old mindset [that] they will come and listen to the doctors and then they will follow (instructions). (Pharmacist 14)

Pharmacists also perceived that some patients tended to be shy during consultations, which was seen as a barrier to their active engagement in consultations.

Some patients are very shy, they definitely won’t ask questions. Sometimes they don’t really want to talk. (Pharmacist 3)

Some patients also agreed that they were more inclined to accept and practice a passive role in healthcare consultations because they perceived that it was not the cultural norm for patients in Malaysia to openly express their opinions.

We still have this mentality where we sit down [and] we listen, then we talk [but] then not really conversing normally… it’s really hard… I’m not saying it’s not possible, but Malaysians are [like] that because they’re not open enough and because they don’t speak up [much]. (Patient 7)

A few patients also perceived that asking questions may be a waste of time during consultations; thus, they would rather just listen passively to the pharmacists.

We just follow what pharmacists say… It’s not that we can’t express our views, it will only be a waste of time, right?…Because everything has been said by them [pharmacists], so we just listen. (Patient 15)

#### Sociocultural characteristics

Pharmacists and patients in this study also mentioned the sociocultural background of patients as a factor that could influence the practice of PCC in consultations. Specifically, the interviewees described the influence of cultural, religious, and language differences, as well as caregiver support, on disease management.

Pharmacists and patients agreed that owing to the diversity in Malaysia, which is attributed to its multiracial and multicultural populations, language, cultural, and religious differences need to be considered during medication decision-making. An example cited was that Muslims in the country may need to alter their medication regimen owing to lifestyle changes during the Ramadan fasting month. Therefore, pharmacists believed that it is important to engage patients during medication consultations to understand their cultural and religious beliefs and how these may influence medication use.

[For example] culture, let’s say if I were to understand a bit more of their belief system, like ‘fasting month’ (Ramadan) for example. Their lifestyles will be changed during the fasting month, and it may affect the way they take medication… (Pharmacist 3)

However, both pharmacists and patients agreed that language differences may be a barrier to communication in medication consultations.

If patients don’t understand my language, then it’s hard to communicate. (Pharmacist 3)

If someone is not comfortable talking in English, then that would be a problem, you know…I think Malaysians are quite diverse in language(s). Sometimes we are not free to express ourselves (because of the language differences). (Patient 7)

In this respect, some pharmacists suggested that a multi-racial healthcare team may be useful in overcoming the language and cultural barriers. However, they acknowledged that it may not always be possible to have pharmacists of different races available at all times.

Support from the family and other caregivers was also cited as an important factor. For example, one patient described the value of having a family member with healthcare experience assisting with the self-management of his disease, including medication intake.

I got a wife [who is] very strict on [me] taking medications because of her profession (as a nurse). She makes sure that I take [the medicines]. Even if it’s 3 hours [after a] missed dose, she will [remind me], “you should take it on time”. (Patient 3)

Although caregivers were described as playing a supportive role, some pharmacists felt that the presence of multiple caregivers may complicate the practice of PCC. An example given was that patients may be more passive in the presence of caregivers who they may not be close or comfortable with, which may affect the treatment planning process. Pharmacists also commented that having several caregivers meant that all caregivers might not be equally engaged with the patient’s care, including their medication management.

…the caregivers might not be the best person to feed the information to. Because some of them, [when] they come, it’s not necessarily the primary caretaker, [and may] be family [members] who only see them once a month. So, the information [received] might not be accurate, which leads to an incorrect (treatment) plan being given. (Pharmacist 1)

### Pharmacist-related factors

#### Personality traits

This factor describes how interviewees thought that the personality traits of pharmacists, such as friendliness, can affect the process of patient-centredness in the consultation. For instance, pharmacists described that pharmacists who are more friendly will make patients feel at ease and hence create an inviting atmosphere for patients to participate in consultations.

It depends on the person (pharmacists) themselves, whether they (pharmacists) are inviting questions or not. If the healthcare provider is someone that is fierce looking, of course, the patients dare not ask questions. Therefore, it just stops there. If you’re more friendly with the patients, you’re more inviting. (Pharmacist 16)

Patients supported this pharmacist’s comment by mentioning that they felt encouraged to ask more questions if the provider was friendly or inviting.

If the providers seemed okay (inviting), then I will talk more, I will ask more questions … If the provider looks serious when telling us that we should not do this, we cannot eat that, this is why our blood sugar is high. That is when I wouldn’t ask further questions, it makes us feel afraid to do so. (Patient 11)

Patients also commented that pharmacists’ demeanours affected their engagement in the consultation. For example, several patients mentioned that pharmacists use a more serious tone or strict demeanour when patients reported non-adherence to their medication schedule.

Some pharmacists here [are] a bit strict. They will be like, very serious and say, you cannot afford to miss your dosage [and] all that. Of course, sometimes [I’m] a bit scared, but then I have to accept it because it’s my responsibility, so they do their part to remind us. (Patient 12)

#### Communication

This factor relates to the exchange of information between pharmacists and patients and describes how verbal and non-verbal interactions could influence the practice of PCC in the MTAC setting. Pharmacists acknowledged the importance of two-way communication between them and patients. However, pharmacists also perceived that many in the profession still practice the biomedical approach, which focuses only on information giving. This leads to a lack of understanding of patients’ views and patients being blamed for not following advice.

I think [that] two-way communication is still very important, which a lot of us fail to do because not everyone can do a two-way communication. Sometimes we [practice] one-way counsel[ling], in the end, we keep blaming [the patients] on why [they] never follow (our advice). (Pharmacist 9)

Patients emphasised the importance of non-verbal communication, such as the tone used by pharmacists. For example, a softer, non-condescending tone was perceived to help patients communicate openly with their pharmacists.

If we want to ask anything, they might scold us; that’s the reason I don’t ask any questions … What they tell us is for our own good, but the way they talk to us [is important]. (Patient 11)

In addition, pharmacists perceived that different providers may be equipped with different levels of communication skills. This may lead to varying levels of PCC activities, such as patient engagement, during consultation with patients. They suggested that communication training with more simulated counselling could be advantageous to equip pharmacists with patient-centred communication skills.

From what I see, actually, we are engaging patients, maybe just [to] a different extent based on different pharmacists who are counselling. So, if we are able to increase the extent [of applying PCC] through training or more case simulations, perhaps then, there will definitely be changes… (Pharmacist 9)

### Healthcare institutional and system-related factors

#### Healthcare setting resources

There are multiple factors related to healthcare setting resources that may affect PCC practice. Time and manpower were frequently mentioned by pharmacists and patients as important resources, wherein a lack of these resources could impede any approach to engage patients and provide adequate patient education. Pharmacists acknowledged that it may not always be possible to address all the specific concerns and issues that the patient may have about their treatment due to limited time and many patients that are waiting to be seen.

…we don’t have that much of manpower because right now only one person doing (is on duty) for each of the MTAC warfarin days. So, it might be affecting our time, whether we can finish on time or not. (Pharmacist 5)

There are patients waiting outside, and there’s pressure, so you don’t have much time for each of them.. You cannot see one patient for 2 hours just because he wants to share his views; [it’s] not so easy. (Pharmacist 9)

Patients also mentioned that it can be difficult for pharmacists to address every patient’s informational needs in the limited time.

They (pharmacists) are unable to give adequate information to patients due to time constraint. At this time, there are many patients, right? It’s not the patients’ fault, it’s due to the big crowd of patients. They (pharmacists) have to take at least 10 to 15 minutes, and that time is not enough to do a lot of explanation. (Patient 8)

With that being said, pharmacists believed in the importance of spending additional time with patients that have complex medical issues requiring intensive medication adjustments and monitoring.

But we may need, or rather we should, spend a bit more time with [patients with] problematic INR (International Normalized Ratio for patients on Warfarin treatment), you know? Those patients with difficult INR control, I think perhaps we can spend more time on that. (Pharmacist 3)

On the other hand, patients commented that they may feel too exhausted to engage in open discussions with pharmacists after long periods of waiting. In addition, the busy setting had caused patients to avoid asking too many questions so as to not affect other patients’ waiting time.

If it’s possible, I don’t want to wait too long. Because of the long wait, my children have to take leave from work to accompany me. Sometimes we wait for the whole day, and then, [when] we enter (the consultation room), we are tired and forget all our questions. (Patient 10)

Another healthcare setting resource highlighted by the participants was patient education aids. Pharmacists believed that more comprehensive patient education aids, such as information leaflets, can be helpful as patients can refer to these aids and use them to self-educate after their consultation.

I think we should provide patients with some leaflets or some counselling references that they can refer [to] at home. We do provide certain ones, but [they are] not comprehensive enough perhaps. So, if we can provide patients with proper guidance… if we can provide them with literature or some aids for them that they can refer to in the future, that will be great. (Pharmacist 14)

Patients also suggested that the use of medication information leaflets could help save time during consultations.

This (MTAC consultation) is like a discussion where 10 minutes are probably enough because they (pharmacists) already provided us leaflets with pictures to educate. So, you do not need an hour, it’s just wasting the time of other people who are waiting, right? (Patient 13)

#### Continuity of care

Continuity of care in the present study relates to patients receiving continuous care from pharmacists with whom they had established long-term professional relationships. Both pharmacists and patients commented that pharmacists seeing the same patient over time would contribute to rapport-building and trust; thus, patients would be more willing to participate in the consultation. In addition, pharmacists believed that patients who are consulted by different and unfamiliar healthcare providers at each follow-up session may avoid disclosing personal concerns.

I believe it’s best to have one pharmacist in a particular portfolio if they are already quite strong in it. Because one thing, patients have more confidence because they know that this person’s been here for so long… So, it’s got that kind of rapport already. Especially for patients who have been long-term in our care. (Pharmacist 13)

Similarly, patients also described being ‘fearful’ or unwilling to be active in the consultation if the pharmacist is someone they just met.

Because we are used to him (the pharmacist) … If every time I come, it’s a different person…we become afraid of asking too much. If I know him for years, then I can always ask questions. (Patient 11)

#### Interprofessional collaboration

In this study, interprofessional collaboration was described as multiple healthcare providers from various disciplines working together with patients and caregivers to optimise the quality of healthcare. Pharmacists commented that interprofessional collaboration is an important aspect of PCC, as multiple members of the healthcare team could improve the delivery of individualised care to the patient. For example, pharmacists mentioned that if there is neither proper collaboration nor communication between physicians or pharmacists from different departments, polypharmacy and medication errors, such as repetition of outdated prescriptions, may ensue.

Patients who come tend to also have appointment with other disciplines… You know, diabetes is a combination of lots of problems. So, sometimes doctors from all these departments, they don’t collaborate well. They don’t communicate well. So, it leads to duplication of medication or repeating old doses of medication. (Pharmacist 7)

Pharmacists also described having multidisciplinary healthcare meetings which also included patients and their caregivers. This approach was perceived to be useful to better understand patients’ personal concerns about their medication and to build a rapport with them.

I think currently we do invite patients…we also have medical team meetings where we invite patients and patients’ family to attend, [so] a multidisciplinary meeting which has pharmacist, dieticians [and] doctors, mainly to get their input on what is troubling them in terms of medication. That shows how much we value participation from patients because without knowing their issues, we cannot solve their problems. (Pharmacist 1)

## Discussion

Patients can benefit from the implementation of a PCC approach by pharmacists in medication management consultations. However, only a few studies have demonstrated how the PCC approach is operationalised in pharmacy settings, especially regarding factors that influence the practicality of this approach. To the authors’ knowledge, this is the first study to investigate the views of both pharmacists and patients on potential barriers and facilitators of PCC in Malaysian hospital pharmacist consultations. This study revealed several factors related to patients, pharmacists, and healthcare institutions and systems, which can either facilitate or constrain the practice of PCC during consultations with pharmacists.

Patient-related factors, such as passive attitudes, were frequently attributed to their lack of knowledge about their health conditions and treatments, as well as perceptions that expressing their opinions is not the cultural norm during consultations. Another study also suggested that patients’ passive behaviour could be reinforced by the providers, whereby passivity equates to “easy” or “best” patients. This desire to be the “good” patient could also be motivated by perceived benefits, such as conflict avoidance or having providers “on your side” [25]. Additionally, patients’ passivity could be attributed to Malaysian patients’ preference to defer decisions to their providers, despite attempts by healthcare providers to practice shared decision-making [12,26]. Although these factors were cited as patient-related, it is noteworthy that healthcare providers play an important role in and are responsible for addressing these barriers. For example, to reduce the patient-provider knowledge gap, pharmacists can improve patients’ medication literacy by providing tailored educational materials and organising patient support groups to boost knowledge exchange among patients [27]. Interventions should also be undertaken to empower patients and increase their involvement in medication decision-making during consultations. Decision aids may be useful in allowing patients to ask questions, express emotional needs, and make healthcare decisions together with their providers [3]. The use of decision aids could facilitate shared discussions on therapeutic plan adjustments (e.g. dose adjustment and lifestyle modifications) in MTAC, which could improve patient self-understanding and confidence in decision-making [13,28,29].

Supportive and respectful communication by pharmacists is also viewed as an important component for successful PCC implementation. Both pharmacists and patients in this study agreed that healthcare providers should portray friendly behaviour and encourage two-way communication to ensure that patients are more actively engaged in the encounter. Patients mentioned that pharmacists with a strict and unfriendly demeanour during consultations may cause a feeling of unease, which can affect their engagement in the encounter. However, some studies also argued that strict behaviours from healthcare providers might be necessary to induce a change in attitude for those patients who are resistant to medical advice [30]. Nonetheless, such behaviours have generally been associated with a declining quality of patient care [31,32]. Notably, strict behaviours conflict with the core aspects of patient-centredness, which promote patient-provider therapeutic relationships and patient engagement [33,34]. One study proposed that pharmacists who underwent a traditional health education model upholding paternalism and technical knowledge might have contributed to such behaviours [35]. This medicine-centred behaviour was described as ‘natural attitude’, whereby pharmacists were focused on correct medicine use rather than adopting a patient-centred approach [4,35]. Accordingly, it was suggested that pharmacists should aim to achieve openness through strategies such as listening to patients, acknowledging patients’ emotional needs, and reflecting on their own behaviours and judgement [4]. In this respect, patient-centred approaches, such as motivational interviewing, may be useful in resolving patient ambivalence, supporting emotional needs, and motivating behavioural changes, including medication adherence [36]. Hence, communication skills training programs for pharmacists should consider incorporating motivational interviewing to enhance patient-centred communication skills among pharmacists [37,38].

Effective communication for patient engagement can be complicated by language and cultural barriers, which were frequently brought up in this study. Malaysia is a diverse country with many ethnicities; thus, Malaysians have varying proficiency in languages and possess different cultural and religious beliefs [39,40]. In fact, studies suggest that some patients prefer to speak in their native languages, which can be difficult for providers who do not speak those languages [41,42]. It has been emphasised that language barriers could complicate patient-provider communication, which in turn leads to poor patient engagement and affects overall health outcomes [14]. Certain approaches can be undertaken by healthcare providers to overcome language and cultural barriers. For example, healthcare providers should try to instil cultural competence, a term used to describe the understanding and awareness of diverse cultural and religious practices. This would allow for a consultation that respects patients’ values and health beliefs, which are aspects that are fitting to the practice of PCC [43]. Interviewees have suggested the hiring of healthcare providers from various ethnicities or who are multilingual; however, this is not always practical in the healthcare setting. Therefore, an approach is needed to promote cultural competence and overcome language barriers, such as attending basic language classes [44], educating healthcare providers about various cultures and religious practices [45], or developing a practical guide that healthcare providers can refer to [46].

Apart from that, patients’ social support, such as from families or other caregivers, could influence active patient engagement. In this study, patients mentioned the effective role of caregivers in helping them manage their medication use. However, pharmacists commented that the presence of caregivers during the encounter might complicate the practice of PCC, particularly in treatment planning or decision-making. Another study supports this finding, adding that caregivers may substantially influence treatment decision-making, which may conflict with the patient’s preferences [47]. Nonetheless, every caregiver involved should be equally engaged because they play a pivotal role as the source of assistance, and individualised care should be provided that is tailored to the needs of patients and their respective families [48,49]. Consequently, healthcare providers should adopt strategies to encourage active engagement from both patients and their respective caregivers. For example, encouraging the sharing of information from both patients and their caregivers, as well as responding to their needs, allows for an open discussion of disease management, which improves the engagement of both caregivers and patients alike [50].

Pharmacists and patients also highlighted the importance of continuity of care (i.e., seeing the same provider at each follow-up) in providing opportunities for the practice of PCC through the establishment of a trusted relationship. Interviewees in this study cited that patients may feel afraid or awkward when seeing different pharmacists at each visit, which may impede rapport-building efforts. Further, patients become reluctant to raise any further inquiries. This finding is similar to that of another study [51]. Pharmacists may also have varying communication skills, which could potentially cause inconsistent care delivery to patients in terms of patient engagement and addressing individual concerns [52]. Studies have found that consistent care provided by the same pharmacists may positively contribute to patients’ medication management, such as reduced adverse drug reactions and rehospitalisation [53]. Nevertheless, pharmacists shared that the practice of continuity of care can be a challenge, especially when there is a shortage of manpower, time constraints, and high patient load. Studies have suggested that the application of PCC through training (e.g. using empathy and patient-centred communication) may save time in the long term. Nonetheless, future research should investigate the effects of PCC training on consultation duration [54]. Interviewees also agreed that the use of educational materials, such as information leaflets or patient decision aids, allows for a more focused and time-saving consultation, a finding which is consistent with that of other studies [55]. Nonetheless, coping with barriers, such as time constraints and inadequate staffing, requires additional support at the organisational level to address these issues [18].

While ensuring continuity of care for patients is beneficial, pharmacists also emphasised the value of interprofessional collaboration in operationalizing PCC in routine healthcare. Pharmacists reported that poor collaboration or inconsistent communication between healthcare providers may lead to duplication of therapy or unnecessary prescription of medicines. In addition, it may cause gaps in care delivery, which may lead to several unclear treatment plans that may be counterproductive to the provision of PCC [56]. Thus, healthcare institutions should implement steps to encourage interprofessional collaboration within multidisciplinary teams for the optimisation of PCC. One solution, as suggested by pharmacists in this study, includes the organisation of interprofessional meetings or conferences that also involve patients and their families. Studies have been found to support this finding, where interprofessional case conferences may facilitate individualised treatment decision-making and promote information sharing [57]. Nevertheless, there are challenges associated with this solution, as time will be needed to implement an interprofessional approach towards PCC in current healthcare teams [58,59]. However, this can be considered for targeted patient groups, such as those with complex medical conditions that require monitoring.

Some limitations in this study need to be acknowledged. First, convenience sampling was employed for patients. Because only willing participants were sampled, this might have introduced selection bias in the data. Second, patients were mainly sampled from either diabetes or warfarin management MTAC; thus, the findings may not be applicable to other MTAC types owing to possible variations in practice. Nevertheless, pharmacists were recruited using a purposive and snowball sampling approach, covering several MTACs across different chronic diseases, which allowed for more comprehensive data coverage for this study. Future studies are recommended to compare the barriers and facilitators of PCC implementation in pharmacy contexts across different healthcare settings (e.g. community pharmacy) and MTAC types (e.g. human immunodeficiency virus).

## Conclusion

The inclusion of pharmacists and patients in this study provided useful insights into the barriers and facilitators of PCC in pharmacist consultations, which are important in the design and implementation of strategies in the local healthcare setting. Pharmacists can support the operationalization of PCC in their practice by using additional support materials, such as educational aids. Patient-centred communication skills training for pharmacists may also be beneficial to ensure that they have better engagements with patients and their respective caregivers. Finally, healthcare institutions can overcome existing barriers through changes at the organisational level and by encouraging interprofessional collaboration that involves patients and their families.

## Supporting information

S1 Appendix Detailed characteristics of individual participants. (PDF)

S2 Appendix Consolidated criteria for reporting qualitative studies (COREQ): 32-item checklist. (PDF)
