# Supplementary material for: Barriers and facilitators to patient-centred care in pharmacy consultations: A qualitative study with Malaysian hospital pharmacists and patients
Source: PLoS One. 2021 Oct 7;16(10):e0258249. doi: 10.1371/journal.pone.0258249 (PMC8496827; doi:10.1371/journal.pone.0258249)
Supplement: S1 Appendix — (PDF) [file pone.0258249.s001.pdf]

S1 Appendix: Detailed characteristics of individual participant

Demographic characteristics of pharmacists

**Pharmacist**	**Gender**	**Age**	**Ethnicity**	**Highest academic qualification**	**Type of MTAC[a] involvement**	**Years of experience as practising pharmacist**	**Hours spent in MTAC[a] consultation per week**
1	Female	25-34 years old	Chinese	Bachelor's degree	Warfarin	5-10 years	5-10 hours
2	Female	25-34 years old	Chinese	Bachelor's degree	Respiratory	<5 years	<5 hours
3	Female	35-44 years old	Chinese	Master’s degree	Warfarin	>10 years	<5 hours
4	Female	35-44 years old	Chinese	Bachelor's degree	Warfarin	>10 years	<5 hours
5	Female	25-34 years old	Indian	Master’s degree	Warfarin	5-10 years	<5 hours
6	Female	25-34 years old	Chinese	Bachelor's degree	Diabetes	5-10 years	5-10 hours
7	Male	25-34 years old	Chinese	Bachelor's degree	Diabetes	5-10 years	5-10 hours
8	Female	35-44 years old	Malay	Bachelor's degree	Psychiatry	5-10 years	<5 hours
9	Female	25-34 years old	Chinese	Bachelor's degree	Diabetes	<5 years	<5 hours
10	Female	25-34 years old	Chinese	Bachelor's degree	Diabetes	5-10 years	<5 hours
11	Female	25-34 years old	Malay	Bachelor's degree	Warfarin	5-10 years	<5 hours
12	Female	25-34 years old	Chinese	Master’s degree	HIV[b]	>10 years	<5 hours
13	Female	25-34 years old	Indian	Bachelor's degree	HIV[b]	5-10 years	5-10 hours
14	Female	35-44 years old	Chinese	Master’s degree	Diabetes	>10 years	<5 hours
15	Female	25-34 years old	Indian	PhD	Warfarin	5-10 years	<5 hours
16	Female	25-34 years old	Chinese	Master’s degree	Warfarin	>10 years	<5 hours
17	Female	25-34 years old	Chinese	Bachelor's degree	Warfarin	<5 years	<5 hours
18	Female	25-34 years old	Chinese	Bachelor's degree	Thalassemia	>10 years	5-10 hours

[a]MTAC: Medication Therapy Adherence Clinic

[b]HIV: Human Immonodeficiency Virus

Demographic characteristics of patients

Patient	Gender	Age	Ethnicity	Education level	Month personal income[a]	Years of follow-up	Type of MTAC[b]
1	Female	55-64	Malay	Secondary school	Not working/ retired	>1 year	Diabetes
2	Female	45-54	Indian	Diploma/ Pre-University	< RM2,000	6-12 months	Haematology
3	Male	Above 65	Chinese	Secondary school	Not working/ retired	>1 year	Warfarin
4	Male	18-24	Malay	Secondary school	Not working/ retired	<6 months	Warfarin
5	Male	55-64	Chinese	Secondary school	Not working/ retired	>1 year	Diabetes
6	Male	25-34	Indian	Diploma/ Pre-University	< RM2,000	>1 year	Warfarin
7	Female	Above 65	Malay	Secondary school	< RM2,000	<6 months	Warfarin
8	Male	45-54	Malay	Secondary school	Not working/ retired	>1 year	Warfarin
9	Female	Above 65	Malay	Primary school	Not working/ retired	>1 year	Warfarin
10	Female	Above 65	Malay	Primary school	Not working/ retired	>1 year	Warfarin
11	Female	45-54	Malay	Secondary school	Not working/ retired	>1 year	Warfarin
12	Female	35-44	Indian	Bachelor's degree	RM5,001-RM8000	>1 year	Warfarin
13	Male	18-24	Malay	Secondary school	Not working/ retired	>1 year	Warfarin
14	Male	55-64	Malay	Bachelor's degree	Not working/ retired	>1 year	Warfarin
15	Male	55-64	Malay	Secondary school	Not working/ retired	>1 year	Warfarin
16	Female	Above 65	Chinese	Secondary school	Not working/ retired	>1 year	Warfarin
17	Female	55-64	Malay	Secondary school	Not working/ retired	>1 year	Warfarin

[a]Monthly personal income in Malaysian Ringgit currency (RM)
[b]MTAC: Medication Therapy Adherence Clinic
