# Supplementary material for: Barriers and facilitators to patient-centred care in pharmacy consultations: A qualitative study with Malaysian hospital pharmacists and patients
Source: PLoS One. 2021 Oct 7;16(10):e0258249. doi: 10.1371/journal.pone.0258249 (PMC8496827; doi:10.1371/journal.pone.0258249)
Supplement: S2 Appendix — (PDF) [file pone.0258249.s002.pdf]

S2 Appendix: COREQ checklist

Developed from:
Tong A, Sainsbury P, Craig J. (2007) Consolidated criteria for reporting qualitative research (COREQ): a 32-item checklist for interviews and focus groups. International Journal for Quality in Healthcare: 19:349–357

	**Domain 1: Research team and reflexivity**		**Page no.**
1.	Interviewer/facilitator	One interviewer (YKN) carried out the interviews.	Page 10
2.	Credentials	The credentials of the researchers are: YKN (BPharm), NMS (PhD), LSL (MSc), LTP (MPharmClin) & WWC (PhD).	
3.	Occupation	YKN was a PhD student at the time of the study while NMS and WWC are lecturers. LSL and LTP are hospital clinical pharmacists.	
4.	Gender	YKN is a male, and the others are female.	
5.	Experience and training	All researchers are experienced qualitative interviewers.	
6.	Relationship established	YKN used to work with a few pharmacists involved in this study for a brief period but only knew each other at the level of acquaintance. No prior relationship between the interviewer and interviewees existed besides that.	
7.	Participant knowledge of the interviewer	Prior to the interview session, the purpose of the interview was explained to the participants and written informed consent was then obtained from all participants.	Page 7
8.	Interviewer characteristics	The participants knew that the interviewer was a student researcher. Sufficient time was spent by the interviewer to ensure familiarisation with the environment and practice setting of each site.	Page 10
	**Domain 2: Study design**		
9.	Methodological orientation and theory	This qualitative study comprised semi-structured interviews with MTAC pharmacists and patients to explore their personal views and experiences on the barriers and facilitators that could affect the implementation of a PCC approach in medication consultations. Interview guides were developed based on the PCC integrative framework by Scholl et al. (2014).	Page 6-7
10.	Sampling	Pharmacists were recruited using purposive sampling based on their expertise and experience in various chronic disease management practices in MTAC services. Patients were recruited by convenience sampling.	Page 6
11.	Method of approach	Participants were mostly approached face-to-face.	Page 7
12.	Sample size	Eighteen pharmacists and 17 patients were interviewed.	Page 11
13.	Non-participation	There were no refusals or dropouts.	

14.	Setting of data collection	Interviews with the pharmacists were conducted in their office rooms or cubicles while ensuring sufficient privacy. Patients were taken to a vacant consultation room or a secluded corner in the waiting area to ensure adequate privacy and minimal distraction.	Page 10
15.	Presence of non-participants	Only the interviewer and interviewees were present during the interview.	Page 10
16.	Description of sample	Detailed information about the interviewees are provided in Appendix S1.	
17.	Interview guide	Topics of interview guide are listed in the main text.	Page 7-10, Table 1
18.	Repeat interviews	No repeat interviews were done.	
19.	Audio/visual recording	All the interviews were audio-recorded.	Page 10
20.	Field notes	Field notes were taken for each interview to support data interpretation.	Page 10
21.	Duration	The interviews lasted between 15 and 55 minutes. The average duration of interviews for pharmacists and patients was approximately 29 and 25 minutes, respectively.	Page 11
22.	Data saturation	Interviews were conducted until data saturation was achieved. Data saturation was achieved when no new themes or codes emerged from the last three interviewees from pharmacists and patients, respectively.	Page 10
23.	Transcripts returned	Transcripts were not returned to interviewees. All audiotapes were transcribed verbatim and checked by the researchers to ensure accuracy of the narratives. In addition, participants were prompted during the interviews if further clarity was required to ensure accurate and reflective answers related to the topic.	
	**Domain 3: Analysis and findings**		
24.	Number of data coders	Two researchers (YKN and WWC) performed the initial coding.	Page 11
25.	Description of coding tree	Figure 1 shows the overview of the coding for the themes and subthemes.	Page 11
26.	Derivation of themes	Interview transcripts for both pharmacists and patients were analysed using the thematic analysis method. First, transcripts were repeatedly read to ensure familiarisation with the data. Thereafter, initial codes were produced by YKN and WWC, and emerging themes and subthemes were subsequently generated based on significant patterns in the codes. New codes were then continually defined, and themes and subthemes were continuously reviewed and refined using the constant comparison approach.	Page 10-11
27.	Software	Atlas.TI (version 7) was used to manage the data.	Page 11

28.	Participant checking	The data findings were not provided to the interviewees. Nonetheless, participants were prompted if further clarity was required to ensure accurate and reflective answers of their personal experiences (page 7). Additionally, the analysed data was crosschecked with the field notes and memos, and consistency was ensured through consensus meetings held between the research team members (page 10).	Page 7, 10
29.	Quotations presented	Participant quotations were presented throughout the results section.	Page 11-26
30.	Data and findings consistent	There was consistency between the data collected and the findings.	Page 11-26
31.	Clarity of major themes	Factors that could facilitate or hinder a PCC approach in pharmacist consultations were divided into three main themes: patient-related factors, pharmacist-related factors, and healthcare institutional and system-related factors. Themes and their respective subthemes are summarised in Figure 1.	Page 11, Figure 1
32.	Clarity of minor themes	Yes, there is a discussion of minor themes.	Page 11-26
